# Experience with Balloon Dilatation in Crohn's and Non-Crohn's Benign Small-Bowel Strictures: Is There a Difference?

**DOI:** 10.1155/2019/1262595

**Published:** 2019-05-06

**Authors:** Akiyoshi Tsuboi, Shiro Oka, Shinji Tanaka, Sumio Iio, Ichiro Otani, Sayoko Kunihara, Ryohei Hayashi, Kazuaki Chayama

**Affiliations:** ^1^Department of Gastroenterology and Metabolism, Hiroshima University Hospital, Hiroshima, Japan; ^2^Department of Endoscopy, Hiroshima University Hospital, Hiroshima, Japan

## Abstract

**Background/Aim:**

Endoscopic balloon dilation (EBD) has been effective for small-bowel strictures in patients with Crohn's disease (CD). However, its efficacy and indication for small-bowel strictures in non-CD patients have not been established. This study evaluated the clinical efficacy and safety of EBD for small-bowel strictures in non-CD patients compared with CD patients.

**Methods:**

Ninety-eight consecutive patients (mean age, 53 years; average observation period, 45 months) with small-bowel strictures diagnosed by double-balloon endoscopy were retrospectively evaluated at Hiroshima University Hospital from August 2003 to April 2017. The average number of procedures, short-term and long-term EBD success rates, and safety profiles between the non-CD and CD groups were examined.

**Results:**

Surgery was selected as the initial treatment in 44 cases (45%) (non-CD group, 27 (61%); CD group, 17 (39%)) as EBD is not indicated. Fourteen non-CD patients had strictures due to malignant tumors, while 13 patients had benign strictures. Twenty-three patients (non-CD, 12; CD, 11) underwent EBD. Forty-three EBD procedures were performed for 17 stricture sites (average: 2.5 procedures/site) in non-CD patients and 41 EBD procedures for 18 stricture sites (average: 2.3 procedures/site) in CD patients. The short-term success rate was 100% (23/23), whereas the long-term success rate was 92% (11/12) in non-CD patients and 82% (9/11) in CD patients. No significant differences in the surgery-free rate occurred between both groups. Furthermore, one adverse event, bleeding after EBD, was encountered in the non-CD group (8%, 1/12).

**Conclusion:**

EBD for small-bowel strictures demonstrated good clinical outcomes in non-CD patients.

## 1. Introduction

Recently, small-bowel diseases such as ulcerations, angiodysplasias, tumors, and strictures can be diagnosed by balloon endoscopy and capsule endoscopy (CE). Particularly, double-balloon endoscopy (DBE) and single-balloon endoscopy have been widely used for the diagnosis and endoscopic treatment of small-bowel diseases. In 2001, Yamamoto et al. [1] first described DBE as a new method to visualize the entire small-bowel. Besides direct observation, DBE allows for histological diagnosis by forceps biopsy and interventional treatment including hemostasis, polypectomy, endoscopic mucosal resection, and balloon dilation. Therefore, DBE has become a key modality for evaluating small-bowel diseases, with a greater diagnostic yield than conventional modalities such as fluoroscopic enteroclysis. Recently, there have been several reports regarding the diagnostic and therapeutic roles of DBE [2–4].

Small-bowel strictures are often caused by chronic inflammatory diseases such as Crohn's disease (CD). In CD patients, the risk of surgery 10 years after a diagnosis of intestinal stricture was 38–55% in a population-based cohort study [5]. Moreover, recurrent strictures often develop after surgical resection. The rate of reoperation was 31.4% and 61.2% in patients who underwent initial surgery within 5 and 10 years, respectively [6]. CD patients with repeated small-bowel surgical resections for strictures are at risk of developing short bowel syndrome. Therefore, an alternative therapeutic approach has been applied to avoid the need for small-bowel resection.

Endoscopic balloon dilation (EBD) has recently been effective in treating small-bowel strictures caused by CD [7]. However, only few reports have evaluated the long-term outcomes of EBD using DBE for small-bowel strictures due to CD [7–9].

The causes of small-bowel strictures in non-CD patients are diverse, including neoplasms, nonsteroidal anti-inflammatory drug- (NSAID-) induced ulcerations, intestinal tuberculosis, enteritis, and postsurgical strictures. Although the utility of EBD in CD has been reported, there are few reports on the efficacy and safety of EBD for small-bowel strictures caused by non-CD conditions, such as strictures caused by NSAID-induced ulcerations [10, 11]. Currently, the diagnostic and therapeutic strategies for small-bowel strictures in non-CD conditions have not been standardized.

This study thus is aimed at evaluating the efficacy and safety of EBD for small-bowel strictures in non-CD patients compared with CD patients.

## 2. Material and Methods

### 2.1. Patients

We retrospectively examined 98 consecutive patients (mean age, 53 years; average observation period, 45 months) with small-bowel strictures, who were among 1,318 patients who underwent DBE from August 2003 to April 2017 at Hiroshima University Hospital. Regardless of the presence of abdominal symptoms from gastrointestinal obstruction, a small-bowel stricture was defined as a lesion wherein an endoscope could not pass through. We excluded the cases that we could not observe for more than 1 year in this study. We performed EBD for small-bowel strictures in 23 patients (non-CD group, 12 patients; CD group, 11 patients). Hence, a total of 23 patients were evaluated in this study.

This study was conducted in accordance with the Declaration of Helsinki and was approved by the Institutional Review Board of the Hiroshima University Hospital (approval number: E-1142, Institutional Review Board registration date: March 23, 2018). All patients were informed of the risks and benefits of EBD and provided written informed consent prior to the procedure. None of the patients refused to undergo EBD for small-bowel strictures during the study period.

### 2.2. Methods

The indications for EBD were as follows: (1) small-bowel strictures causing obstructive symptoms or proximal extension from the stricture site as shown by diagnostic imaging (fluoroscopic examination or computed tomography), (2) benign strictures wherein an endoscope could not pass through, (3) stricture length ≤ 5 cm, and (4) strictures without a fistula, abscess, deep ulceration, severe adhesion, or curvature [8].

Our management for small-bowel strictures was as follows. In cases of small-bowel stricture due to malignant tumor, we selected surgical resection or bypass considered by general condition. In cases of benign small-bowel stricture, we selected EBD as the first choice when the above adaptation was satisfied. We selected surgery if patients did not satisfy the EBD indication. In asymptomatic cases of benign small-bowel strictures, we selected medical treatment or follow-up.

**Figure 1:**
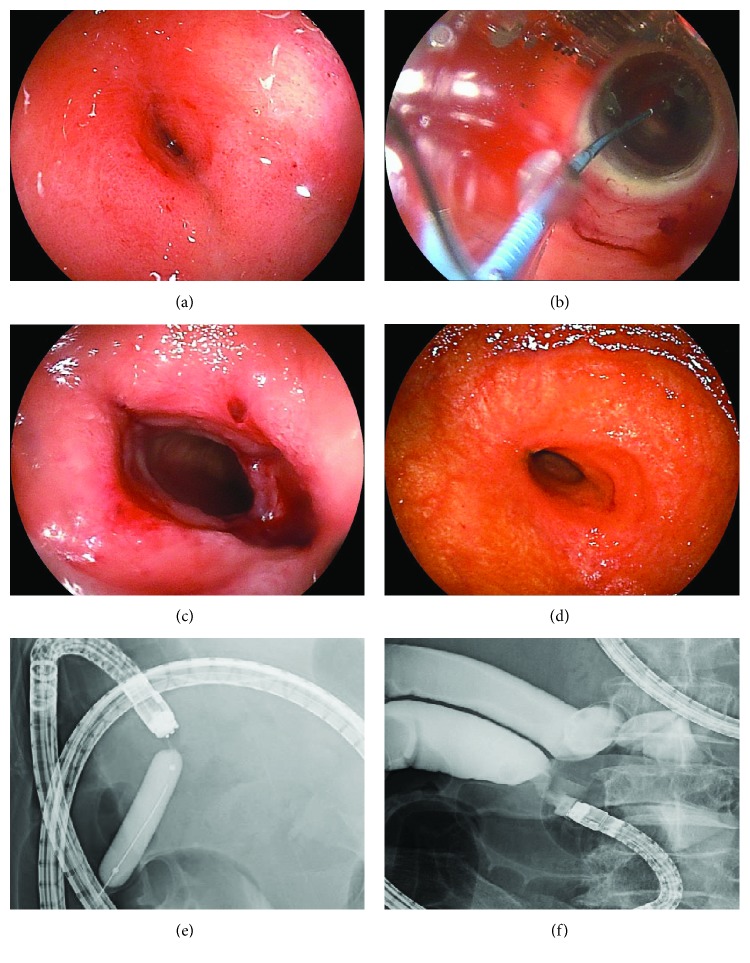
The endoscopic balloon dilation procedure. (a) Endoscopic imaging of a stricture due to scar after chemotherapy for malignant lymphoma. (b) Balloon dilation. (c) After dilation. (d) Endoscopic imaging of stricture after 5 times of dilation. (e) Contrast study of the stricture during dilation. (f) Contrast study of the stricture after dilation.

The patients underwent overnight fasting in preparation for EBD. In principle, we selected the antegrade approach for the procedure. EBD was carried out using a DBE (EN-450 T5, EN-580 T5; Fujifilm Medical Co., Tokyo, Japan) and an 8–18 mm through-the-scope (TTS) balloon catheter (CRE™; Boston Scientific Co., Natick, MA, USA) measuring 7.5 Fr and 5.5 cm in length. The size of the balloon was determined according to the size of the stricture site. The balloon was positioned across the stricture and filled with diluted Gastrografin and was inflated to a pressure of 1–8 atm for 30 s. Dilation was performed by monitoring the pressure of the inflated balloon using a dilator under X-ray guidance. The maximum dilation diameter and balloon pressure were confirmed by fluoroscopy and determined at the discretion of the operating endoscopists. After dilation, we performed a small-bowel follow-through using Gastrografin to confirm leakage outside the intestinal tract (Figure 1).

We evaluated the average number of procedures, short-term and long-term EBD success rates, and safety profiles between the non-CD and CD groups. Complications were defined as perforation and active bleeding requiring surgery or blood transfusion after EBD. Short-term EBD success was defined as the disappearance of abdominal symptoms due to gastrointestinal obstruction and long-term EBD success as having no surgery for >1 year. Oral-side intestinal extension was defined as existence of a clear stricture of the small-bowel toward the oral side by computed tomography or transabdominal ultrasonographic examination. Meanwhile, endpoint of EBD was defined as successfully passing the endoscope through the stricture site. In our hospital, EBD was performed repeatedly when an endoscope could not pass through the stricture site. Redilation was performed when abdominal symptoms recur due to gastrointestinal obstruction.

### 2.3. Statistical Analyses

The chi-squared test or Fisher's exact test was used for comparison of frequencies. The Kaplan-Meier method and log-rank test were used for analyzing the cumulative surgery-free rate. A *P* value of <0.05 was regarded as statistically significant. The software program JMP Pro 13 (SAS, Cary, NC, USA) was used for the statistical analyses.

## 3. Results

### 3.1. Cohort Details

**Figure 2:**
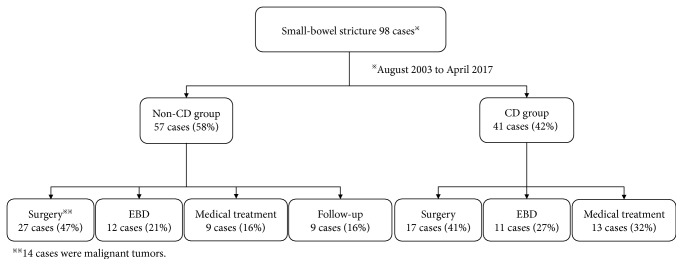
Initial treatment for small-bowel strictures. Surgery was selected as the initial treatment in 27 cases of the non-CD group (47%) and 17 cases of the CD group (41%). In total, surgery was selected as the initial treatment in 44 cases (45%). In the surgery cases of the non-CD group, 14 patients (52%) had strictures due to malignant tumor. EBD was performed in 23 cases (23%) overall as the initial treatment. Twelve cases (21%) and 11 cases (27%) had EBD performed as the initial treatment in the non-CD and CD groups, respectively EBD: endoscopic balloon dilation.

Figure 2 shows the details of initial treatment in this study. We selected surgery as the initial treatment in 44 cases (non-CD group, 27 cases; CD group, 17 cases) and performed EBD as the initial treatment in 23 cases (non-CD group, 12 cases; CD group, 11 cases).  Table 1 shows the clinical characteristics of all cases. Non-CD patients were significantly older than CD patients. The CD group had a higher proportion of males. Moreover, the CD group had a significantly higher proportion of multiple strictures. Although the CD group tended to have more stricture sites, there was no significant difference between both groups. The CD group had a significantly higher number of stricture sites located in the distal small-bowel, while the non-CD group had significantly more sites in the proximal small-bowel. In the cases of medical treatment in the non-CD group, their etiologies were intestinal tuberculosis. No case required additional surgery among the follow-up cases. The primary diseases of all cases and all surgery cases in the non-CD group are shown in Tables 2 and 3, respectively. The non-CD surgery cases included 14 cases of small-bowel strictures due to malignant tumors. Among them, 11 cases were malignant strictures due to primary small-bowel cancer.

### 3.2. Technical Details

We performed EBD for 12 cases of small-bowel strictures in the non-CD group. The primary diseases of these EBD cases are shown in Table 4. These EBD cases included three cases of intestinal tuberculosis, three cases of NSAID-induced ulceration, two cases of a complete response after chemotherapy for malignant lymphoma, two cases of ischemic enteritis, and two other cases. In the CD group, one patient underwent EBD after surgery due to recurrence of stricture. We performed EBD for a total of 11 cases of small-bowel strictures in the CD group.

### 3.3. Outcomes and Complications

**Figure 3:**
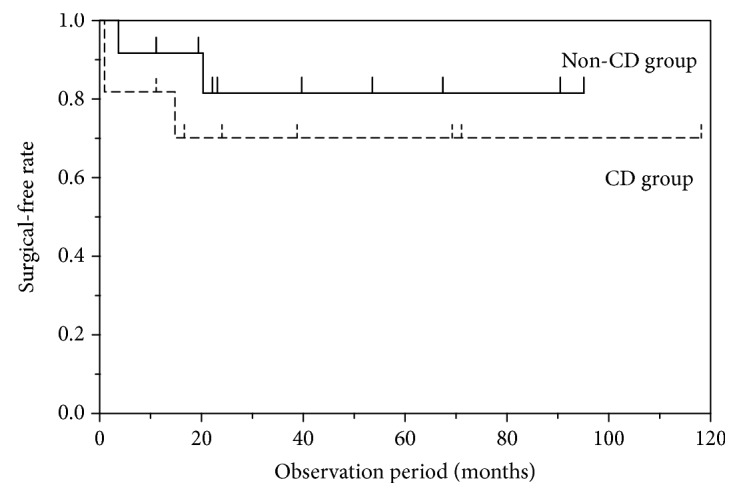
The cumulative surgery-free rate. The cumulative surgery-free rate of EBD cases after 1 year and 2 years from the initial EBD in the non-CD and CD groups was 92% (11/12) and 83% (10/12) and 82% (9/11) and 73% (8/11), respectively. There were no significant differences in the surgical-free rate between the non-CD and CD groups.

All EBD procedures were performed successfully in both CD and non-CD groups. All 23 patients had confirmed disappearance of abdominal symptoms after EBD. Thus, the short-term success rate was 100% in both groups. Five patients underwent surgery during the observation period: two patients in the non-CD group (17%, 2/12) and three patients in the CD group (27%, 3/11). In the additional surgery cases in the non-CD group, the etiologies of small-bowel strictures were intestinal tuberculosis and circumferential small-bowel ulceration due to intraperitoneal band. The cases received repeated EBD, but their symptoms did not improve. Therefore, we selected surgery. The reasons for surgery were as follows: recurrent abdominal symptoms resistant to medical treatment or EBD (non-CD group, two cases; CD group, one case) and remaining strictures that could not be approached by DBE (CD group, two cases). Two patients (1 from each group) underwent surgery >1 year after initial EBD. The long-term success rate was 92% (11/12) and 82% (9/11) in the non-CD and CD groups, respectively. As shown in Figure 3, no significant differences in the surgery-free rate occurred between both groups.

Bleeding occurred in only one case in the non-CD group (8%) after EBD for stenosis, due to scar after ML chemotherapy. In this case, EBD was performed repeatedly because of recurrent symptoms of abdominal obstruction. After four EBD procedures, the patient had a melena and progression of anemia. Although the patient required blood transfusion, the bleeding was stopped by conservative treatment including fasting and infusion of hemostatic drugs. No complications were found in the CD group.

Table 5 shows a comparison of clinical outcomes of EBD between both groups. In summary, the average number of times that EBD was performed was 2.5 (43 EBD procedures for 17 stricture sites) in the non-CD group and 2.3 (41 EBD procedures for 18 stricture sites) in the CD group. There were no significant differences in the number of procedures, short-term success rate, long-term success rate, surgery avoidance rate in the observation period, and complications between both groups.

## 4. Discussion

This study investigated the therapeutic management of small-bowel strictures with EBD in both non-CD and CD patients. With the development of the endoscope, we can easily observe the small-bowel using DBE. DBE can be used for histological diagnosis by forceps biopsy and for endoscopic treatment of small-bowel disease [12]. In clinical practice, the etiology of a small-bowel stricture can be diagnosed by enteroscopy and histology. Moreover, surgery should be the curative or palliative treatment for malignant stricture. Based on our findings, EBD showed good clinical outcomes for small-bowel strictures in the non-CD group regardless of etiology; thus, EBD may be considered for benign strictures in a non-CD patient regardless of etiology.

Recently, a few cohort studies reported that EBD can be an alternative treatment to surgery for small-bowel strictures in CD [6, 8, 9, 13–16]. Hirai et al. [7] performed the largest cohort study on short-term and long-term clinical outcomes of EBD for small-bowel strictures in CD. They reported a short-term success rate of 80% and a cumulative surgery-free rate of 79% at 2 years and 73% at 3 years. EBD using DBE was unsuccessful in 13 of 65 cases (20%): the endoscope could not be inserted up to the stricture site in 8 cases and the guidewire or balloon could not be maintained at the correct position of the stricture in 5 cases. They also reported that successful EBD cases showed significantly higher surgery-free rates than unsuccessful cases using the Kaplan-Meier method. In a systematic review of 13 published articles, Baars et al. [17] reported on the efficacy and safety of EBD for small-bowel strictures in both CD and non-CD cases. In their study, the average follow-up time was 31.8 months per patient and the complication rate was 4.8% per patient. During the follow-up period, EBD (defined as nonsurgical treatment) was performed in 80% of patients. Meanwhile, in our study, we revealed the efficacy and safety of EBD for benign small-bowel strictures in non-CD patients.

To our knowledge, there are only few cohort studies or case reports on the efficacy and safety of EBD for strictures in non-CD patients [10–12, 14, 18–21]. Furthermore, the clinical outcome of small-bowel strictures in non-CD patients based on long-term observation remains unclear. We considered that the first treatment of choice for malignant stenosis is surgery, including resection or bypass for palliative treatment. Indeed, we selected surgical treatment for malignant small-bowel strictures at 88% (14/16). The remaining two cases were unable to endure an operation because of poor general conditions. Recently, endoscopic metallic stent placement has been performed as palliative treatment for malignant stenosis [22–24]. For benign strictures, however, the treatment has not been standardized and there is currently no consensus on whether surgical or medical treatment is more appropriate. This could be attributed to the diverse etiology of small-bowel strictures, including NSAID-induced ulceration, intestinal tuberculosis, ischemic enteritis, and idiopathic causes.

A few cases of EBD for small-bowel strictures due to NSAID-induced ulceration, one of the representative conditions causing gastrointestinal strictures in non-CD patients, have been reported [10, 11, 25]. Small-bowel injury due to NSAIDs was reported as “diaphragm disease” [26]. Diaphragm disease is characterized by a pinhole lumen of 2–3 mm in diameter and a thin diaphragm. The risk of perforation with EBD for diaphragm disease could be low [27]. Intestinal tuberculosis may occur with gastrointestinal obstruction. The gastrointestinal obstruction may also be exacerbated during antituberculosis treatment due to healing by cicatrization [28]. It was reported that about 20–40% of patients with abdominal tuberculosis presented with an acute abdomen and required surgical management [29]. The experience of EBD in patients with ileal tuberculosis is limited to a few case reports [21, 30, 31]. Ischemic enteritis can result in complete healing, chronic enteritis, or stricture [32]. In patients with strictures due to ischemic enteritis, there was only one case series by Nishimura et al. [33]. Moreover, the mean length of the stenosis tends to be longer than that seen in cases of CD [34]. Hayashi et al. [25] performed EBD in seven cases due to ischemic enteritis, and three cases eventually underwent surgery. Small-bowel ML may also result in gastrointestinal complications such as perforation, bleeding, and ileus. The frequency of perforation, bleeding, and ileus is 7–17% [35–38], 4–38% [39–41], and 6–18% [38, 41], respectively. ML is known to develop not only before treatment but also after the treatment. While stricture formation might be considered a predictable complication of primary small-bowel ML, it has not been identified in the previous studies [39, 42, 43]. There were some case reports on EBD for small-bowel strictures occurring after or during chemotherapy for primary small-bowel ML [44–47]. Cho et al. reported a case of perforation after EBD for an intestinal stricture due to ML.

Our study revealed that the observation period was significantly longer in the CD group. CD patients are required long-term follow-up because of repetition of relapse and remission. On the other hand, it is rare to repeat relapse by eliminating the causes in non-CD cases of benign small-bowel strictures, such as NSAID-induced ulceration. There are many benign strictures, and their clinical backgrounds seem to have difference in the observation period in this study.

This study has some limitations. First, it is a single-center retrospective study. The retrospective design could have resulted in recruitment bias. Second, the number of participants was relatively small. Hence, further large prospective cohort studies will help evaluate the key predictors of long-term EBD success. Third, small-bowel strictures were caused by several etiologies. Although we examined various etiologies of small-bowel strictures in the non-CD group, the list remains limited. Lastly, the procedures were not performed according to a defined study protocol. Balloon diameter, interval between dilations, length of follow-up, or technical approach may vary even between patients analyzed in the same study. Therefore, large cohort studies that evaluate the long-term results of EBD according to each etiology and follow a defined endoscopic approach are necessary.

## 5. Conclusions

EBD is a safe and effective treatment for small-bowel strictures in both non-CD and CD patients. In cases of benign small-bowel strictures, EBD was an effective treatment regardless of the etiology. However, it is necessary to prospectively observe a larger number of patients for a longer period to confirm these results more precisely.

## Figures and Tables

**Table 1:** Clinical characteristics of all cases.

Variables	Groups		*P* value
	Non-CD group, *n* = 57 (%)	CD group, *n* = 41 (%)	
Sex			
Male	30 (53)	35 (85)	<0.01
Age (years)	61 ± 1.8	41 ± 2.1	<0.01
Observation period (months)	35 ± 5.3	57 ± 6.4	<0.01
Number of strictures	1.6 ± 1.4	2.1 ± 1.3	0.09
Multiple strictures^∗^	17 (30)	22 (54)	<0.01
Site of stricture			
Upper	16 (28)	2 (5)	<0.01
Middle	23 (40)	9 (22)	0.08
Lower	18 (32)	30 (73)	<0.01
Treatment			
Surgery	27 (47)	17 (41)	0.68
EBD	12 (21)	11 (27)	0.63
Medical treatment	9 (16)	13 (32)	0.09
Follow-up	9 (16)	0 (0)	<0.01

Categorical data are expressed as numbers (%), and quantitative variables as means (standard deviation). ^∗^Multiple strictures were defined as two or more strictures. EBD: Endoscopic balloon dilation.

**Table 2:** Primary diseases of the small-bowel strictures in the non-Crohn's disease group.

Primary disease	Non-CD group, *n* = 57 (%)
Malignant stenosis	16 (28)
Primary small-bowel cancer	12 (21)
Peritoneal dissemination from other organs	1 (2)
ML	2 (4)
Gastrointestinal stromal tumor	1 (2)
Benign stenosis	41 (72)
Intestinal tuberculosis	13 (23)
Ischemic enteritis	7 (12)
Ulceration of unknown origin	7 (12)
Radiation enteritis	3 (5)
NSAID-induced ulceration	3 (5)
Adhesion ileus	3 (5)
Scars after chemotherapy for ML	2 (4)
Others	3 (5)

NSAID: nonsteroidal anti-inflammatory drug; ML: malignant lymphoma.

**Table 3:** Primary diseases of surgery cases in the non-Crohn's disease group.

Primary disease	Non-CD group, *n* = 27 (%)
Malignant stenosis	14 (52)
Primary small-bowel cancer	11 (41)
Peritoneal dissemination from other organs	1 (4)
Malignant lymphoma	1 (4)
Gastrointestinal stromal tumor	1 (4)
Benign stenosis	13 (48)
Ischemic enteritis	4 (15)
Radiation enteritis	3 (11)
Adhesion ileus	3 (11)
Intestinal tuberculosis	1 (4)
Anastomotic ulceration	1 (4)
CEAS	1 (4)

CEAS: Chronic enteropathy associated with SLCO2A1.

**Table 4:** Primary diseases of cases that underwent endoscopic balloon dilation (EBD) in the non-Crohn's disease group.

Primary disease	EBD cases, *n* = 12 (%)
Intestinal tuberculosis	3 (25)
NSAID-induced ulceration	3 (25)
Scars after chemotherapy for ML	2 (17)
Ischemic enteritis	2 (17)
Others	2 (17)

NSAID: nonsteroidal anti-inflammatory drug; ML: malignant lymphoma.

**Table 5:** Outcomes of endoscopic balloon dilation between the non-Crohn's disease (CD) group and CD groups.

Outcomes	Non-CD group, *n* = 12	CD group, *n* = 11	*P* value
Average number of procedures	2.5	2.3	0.62
Short-term success rate	100% (12/12)	100% (11/11)	1.00
Long-term success rate	92% (11/12)	82% (9/11)	0.59
Surgery avoidance rate in the observation period	83% (10/12)	73% (8/11)	0.64
Complication	8% (1/12)	0% (0/11)	1.00
Bleeding	8% (1/12)	0% (0/11)	1.00
Perforation	0% (0/12)	0% (0/11)	1.00

## Data Availability

The data used to support the findings of this study are available from the corresponding author on reasonable request.
